# Role of Galectin-3 in Bone Cell Differentiation, Bone Pathophysiology and Vascular Osteogenesis

**DOI:** 10.3390/ijms18112481

**Published:** 2017-11-21

**Authors:** Carla Iacobini, Claudia Blasetti Fantauzzi, Giuseppe Pugliese, Stefano Menini

**Affiliations:** Department of Clinical and Molecular Medicine, La Sapienza University, 00185 Rome, Italy; carla.iacobini@uniroma1.it (C.I.); claudia.blasettifantauzzi@uniroma1.it (C.B.F.); stefano.menini@uniroma1.it (S.M.)

**Keywords:** galectin-3, osteoblasts, osteoclasts, bone remodeling, vascular osteogenesis

## Abstract

Galectin-3 is expressed in various tissues, including the bone, where it is considered a marker of chondrogenic and osteogenic cell lineages. Galectin-3 protein was found to be increased in the differentiated chondrocytes of the metaphyseal plate cartilage, where it favors chondrocyte survival and cartilage matrix mineralization. It was also shown to be highly expressed in differentiating osteoblasts and osteoclasts, in concomitance with expression of osteogenic markers and Runt-related transcription factor 2 and with the appearance of a mature phenotype. Galectin-3 is expressed also by osteocytes, though its function in these cells has not been fully elucidated. The effects of galectin-3 on bone cells were also investigated in galectin-3 null mice, further supporting its role in all stages of bone biology, from development to remodeling. Galectin-3 was also shown to act as a receptor for advanced glycation endproducts, which have been implicated in age-dependent and diabetes-associated bone fragility. Moreover, its regulatory role in inflammatory bone and joint disorders entitles galectin-3 as a possible therapeutic target. Finally, galectin-3 capacity to commit mesenchymal stem cells to the osteoblastic lineage and to favor transdifferentiation of vascular smooth muscle cells into an osteoblast-like phenotype open a new area of interest in bone and vascular pathologies.

## 1. Introduction

Galectin-3 is a 29- to 35-kDa protein belonging to the family of the β-galactoside binding animal lectins and is constitutively expressed in various tissues, including the bone [1]. It is the only chimera-type galectin in vertebrates and comprises one conserved carbohydrate recognition domain linked to a non-lectin domain through a collagen-like linker region [2]. This lectin has been recognized over the past two decades as being involved in many physiological and pathological processes [2,3]. In quiescent cells, galectin-3 shows prominent cytoplasmic localization, whereas it is found predominantly in the nucleus of replicating cells [3]. Galectin-3 is also secreted into the extracellular space through a non-classical secretory pathway [4]. Here, it interacts with the β-galactoside residues of several glycoproteins, thus forming higher order supramolecular structures resulting in galectin-ligand lattices on the cell surface [5]. These lattices have been shown to play a major role in the regulation of receptor clustering, endocytosis and signaling, thereby controlling important cell functions such as cell transdifferentiation, migration, and fibrogenesis [5,6]. As a component of the cell surface lattice, galectin-3 also regulates the biogenesis of a subpopulation of clathrin-independent carriers involved in the endocytosis of specific cargo proteins, which could represent one of the main mechanisms behind its functions [7].

Intracellular galectin-3 has been implicated in several basic cellular processes related to control of cell differentiation, growth, and apoptosis, as well as in specific cell biosynthetic activities. Galectin-3 has been found in the spliceosome, where it is a required factor in the splicing of nuclear pre-mRNA [8]. This lectin also regulates cell cycle by modulating the activity of cyclins and their inhibitors, as well as the phosphorylation status of retinoblastoma protein [9]. Of great interest for bone biology, intracellular galectin-3 is a key regulator of the Wnt/β-catenin signaling pathway, both through its interaction with β-catenin and because of its structural similarities with it [10]. Intracellular galectin-3 has also been shown to promote cell proliferation [11,12] and favor survival by its anti-apoptotic activity, which is related to its sequence homology and association with bcl-2 [11,13]. However, depending on the cell type, galectin-3 can also promote apoptosis, as demonstrated by its involvement in T-cell and neutrophil death [14].

Extracellular galectin-3 also participates in the control of cell cycle and division. Growth factors immobilization into the galectin-3 lattice is, in fact, an additional mechanism for the regulation of cell growth and differentiation [15]. In addition, cell surface galectin-3 has been shown to regulate cell adhesion in opposite fashions, by both promoting homo- and heterotypic cell-to-cell interactions [16,17] and down-regulating cell adhesion to the extracellular matrix component laminin, thus producing an anti-adhesive effect [18,19].

Another important function of galectin-3 is the uptake and removal of advanced glycation endproducts (AGEs) [20]. AGEs are a heterogeneous class of nonenzymatically glycated proteins, lipids and nucleic acids, which accumulate in tissues during aging and, at a faster rate, in metabolic disorders such as diabetes and obesity [20]. AGEs are toxic molecules inducing tissue injury by direct and indirect mechanisms. In fact, they can exert detrimental direct physicochemical effects by interacting with several molecules, thus inducing changes in enzymatic activity, ligand half-life, binding, and immunogenicity [21]. Moreover, AGEs display indirect deleterious effects by binding to several cell surface receptors, of which the most studied is the receptor for AGEs (RAGE), a 35-kDa member of the immunoglobulin superfamily of receptors [22]. RAGE ligation is associated with cellular oxidative stress [23] and activation of proinflammatory signaling pathways, eventually culminating in tissue inflammation and fibrosis as well as in cell damage and death [20,24]. Noteworthy, galectin-3 and RAGE appear to exert opposite actions as AGE-receptors, with RAGE mediating the injurious effects of AGEs and galectin-3 playing a protective role by favoring removal and degradation of these toxic by-products [2,25].

Finally, galectin-3 plays an important role in the modulation of the immune/inflammatory response, as evidenced by the numerous scientific reports showing a regulatory activity on both innate and adaptive immunity [26]. Galectin-3 has been shown to exert both pro-inflammatory actions, prevailing in an acute setting, and anti-inflammatory effects, particularly in chronic inflammatory conditions, as recently reviewed [20]. In detail, well-established effects of galectin-3 on immune cells include the stimulation of T-cell apoptosis [27], inhibition of T-cell growth and T-helper 1 differentiation [28], down-regulation of T-cell receptor-mediated T-cell activation [29], and induction of alternative (M2) macrophage activation [30].

**Figure 1:**
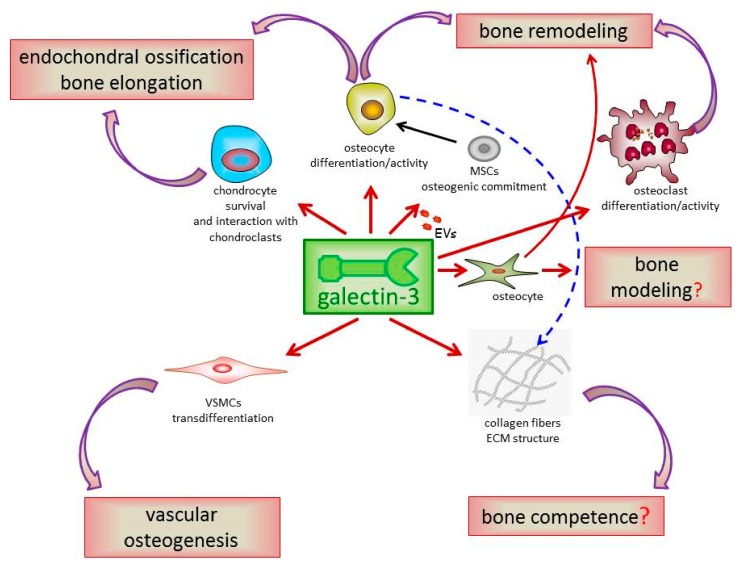
Galectin-3 osteogenic activities in bone and vascular tissue. Galectin-3 is considered a marker of chondrogenic and osteogenic cell lineages and is up-regulated in conjunction with other osteogenic markers during differentiation of bone cells. Consistent with a role in bone physiology, galectin-3 deficiency affects endochondral ossification and bone remodeling. Galectin-3 is also critical for maintaining proper extracellular matrix (ECM) structure and, possibly, bone competence. Being also expressed by osteocytes, it is possible to hypothesize its participation in the regulation of mechanosensory function of these cells and promotion of bone modeling and remodeling also through this mechanism. Finally, galectin-3 activity is central for vascular smooth muscle cells (VSMCs) transdifferentiation into an osteoblast-like phenotype and vascular osteogenesis. EVs = extracellular vesicles; MSCs = mesenchymal stem cells.

As much as concerning the bone, galectin-3 has been shown to be a marker of both chondrogenic and osteogenic cell lineages. Aubin et al. were the first to report that chondroblasts and osteoblasts share the expression of several molecules, including markers such as galectin-3 [31]. Later on, other groups confirmed and extended this initial observation showing that galectin-3 is also found in osteoblasts and osteocytes of the woven trabecular and cortical bone as well as in osteoclasts at the front of ossification and mononuclear cells within bone marrow cavities [1]. In developing and mature bone, galectin-3 expression seems to be restricted to the cytoplasm of chondrocytes and bone cells, although it is occasionally detected in the nuclei of dense non-hypertrophic chondrocytes in the zone of calcification and of young osteoblasts; conversely, no extracellular galectin-3 is detected in developing or mature bone tissue [1]. Noteworthy, Stock et al. demonstrated that the skeletal expression pattern of galectin-3 overlaps at many sites with that of Runt-related transcription factor 2 (RUNX2), which is a key regulator of osteoblast differentiation and chondrocyte maturation [32]. In summary, these early studies showed that galectin-3 is widely distributed both in the developing and mature bone and that its expression is under control of the master regulator of bone growth RUNX2. These findings suggest that galectin-3 may be a key player in all stages of bone biology, thus deserving to be thoroughly investigated as a potential target in bone disorders. In this extensive review, we will present the results of studies that investigated the role of galectin-3 in bone cell differentiation and function, in bone development and remodeling, and in the pathogenesis of inflammatory bone diseases. We will also outline problems and aspects that have not been addressed yet, or addressed only partially, which may represent relevant future research directions. Finally, the role of galectin-3 in vascular osteogenesis, which was recently reported by us and other investigators [33,34,35], will also be discussed. Figure 1 and Table 1 recapitulate the main findings discussed in this review, as well as topics deserving further investigation in the future.

## 2. Galectin-3 in Chondrocyte Differentiation and Endochondral Bone Formation

Endochondral ossification is an essential process by which most of the bones, particularly long bones, of the mammalian skeletal system are formed during embryonic development. At variance with intramembranous ossification, endochondral ossification involves formation of cartilage elements (chondrocytes) from mesenchymal condensation, secretion of extracellular matrix by mature chondrocytes and blood vessels invasion. During bone growth, cartilage anlage is progressively replaced by bone tissue in a highly coordinate process, which is achieved also through a tight balance between the rates of chondrocyte proliferation and apoptosis at the growth plate cartilage. This is a specialized developmental tissue located at the metaphyseal level of long bones in which chondrocytes are organized in three characteristic zones: proliferative, mature, and hypertrophic. Chondrocytes are responsible for the synthesis of the major constituents of the matrix and the enzymes that degrade cartilage matrix. Hence, their function is essential to regulate cartilage synthesis and degradation, thereby coordinating the timing of vascular invasion and subsequent bone deposition [51]. The elongation process takes place through a series of coordinated and complex events, which include apoptosis of the chondrocytes in the terminal hypertrophic zone, partial degradation of the calcified cartilage matrix, and bone matrix deposition by osteoblasts on the remaining cartilage septa, which serve as a scaffold [52]. Failure in any of these events, including perturbations in chondrocyte hypertrophy, would severely affect endochondral ossification and, consequently, normal bone development [53].

Galectin-3 has been initially involved in the process of endochondral bone formation as a regulator of chondrocyte survival. In fact, high levels of galectin-3 protein were demonstrated in the differentiated chondrocytes of the metaphyseal plate cartilage of long bones of both fetal and neonatal mice. The highest concentrations of galectin-3 were found in the cytoplasm of mature and early hypertrophic chondrocytes [1]. At variance, very little expression was detected in the late hypertrophic chondrocytes undergoing terminal maturation and cell death in the zone of calcification, both at protein and mRNA levels. These findings are consistent with the anti-apoptotic function of galectin-3 [11,13,54] and with the observation that galectin-3 null (*Lgals3*^−/−^) mice show accelerated terminal differentiation and death of chondrocytes [1,36]. In addition to chondrocytes, Colnot et al. reported high concentrations of galectin-3 also in the cytoplasm of osteoblasts, osteocytes, and osteoclasts of trabecular and cortical bone, and in monocytes in the bone marrow cavity, thus suggesting a role for this lectin in the differentiation and/or activity of all the cell types present in mature bone [1].

Subsequent comprehensive histological and ultrastructural analyses performed by the same group identified a number of abnormalities in epiphyseal femurs and tibias of fetal *Lgals3*^−/−^ mice, affecting both chondrocytes of the proliferative, mature, and hypertrophic zones and the extracellular matrix of the hypertrophic zone. The greatest abnormalities were detected at the chondrovascular junction, where premature and increased cell death was associated with, and might account for, the increased empty lacunae observed in mutant mice. Consistently, more numerous condensed chondrocytes exhibiting characteristic signs of apoptosis were found in the late hypertrophic zone, indicating that the rate of chondrocyte death was increased in *Lgals3*^−/−^ mice. Taken together, these findings suggest a role for galectin 3 as a regulator of chondrocyte survival and indicate that galectin 3 may also participate in the coordination between chondrocyte death and metaphyseal vascularization. At histological examination, the process of cartilage matrix mineralization appeared to be disturbed in *Lgals3*^−/−^ mice, both in terms of quantity and quality. However, despite these numerous abnormalities of the metaphyseal growth plate cartilage, X-ray analysis conducted in adult *Lgals3*^−/−^ mice did not reveal significant macroscopic anatomical differences (i.e., final size) of long bones, indicating that the initial defects are transient or, alternatively, have no impact on the process of bone elongation [42].

Another mechanism by which galectin-3 may regulate the process of endochondral ossification emerged from studies investigating the cartilaginous bar of the mandibular arch (Meckel’s cartilage), the middle portion of which is known to degrade via hypertrophy and death of chondrocytes and cartilage resorption by chondroclasts/osteoclasts [37]. Sakakura et al. showed that galectin-3 levels were especially higher in hypertrophic chondrocytes adjacent to chondroclasts. This observation suggests that galectin-3 may also coordinate the resorption of calcified cartilage through cell-to-cell interaction with chondroclasts, which are responsible for the synthesis and secretion of matrix metalloproteinases (MMPs) involved in extracellular matrix degradation and in growth plate angiogenesis [37]. Therefore, in addition to controlling chondrocyte cell cycling and survival, evidence suggested that galectin-3 might participate in endochondral bone formation also through regulation of MMPs activity, or vice versa [38,39]. According to this interpretation, deficiency of MMP-9 was shown to induce accumulation of galectin-3 in the expanded hypertrophic cartilage of developing bone, which was associated with a decreased rate of chondrocyte apoptosis and accumulation of late hypertrophic chondrocytes in the same zone [40]. This finding is consistent with the observation that galectin-3 is an endogenous substrate of MMP-9, as indicated by in vitro experiments showing that treatment of wild-type embryonic metatarsals with full-length galectin-3, but not MMP-9-cleaved galectin-3, was able to reproduce the embryonic phenotype of *Mmp-9*^−/−^ mice, characterized by an expanded hypertrophic zone [40]. Altogether, these early studies indicated that galectin-3 and MMP-9 represent two members of the same pathway implicated in the regulation of hypertrophic chondrocyte clearance, degradation of extracellular matrix, and vascular invasion.

## 3. Galectin-3 in Bone Cell Differentiation and Function and Bone Homeostasis

### 3.1. Galectin-3 in Osteoblast Biology and Pathology

Osteoblasts are specialized, not terminally differentiated, mononuclear cells involved in secretion and mineralization of the bone matrix, thus being responsible for new bone formation [55]. Osteoblasts also control bone resorption through regulation of osteoclast activity, thus having a primary role in modeling and preserving skeletal architecture [56]. Osteoblasts derive from the same mesenchymal stem cell (MSC) precursors from which originate chondrocytes, adipocytes, and myoblasts [57], depending on which transcription factor is expressed [55,57,58]. Among the most important transcription factors recognized as specific and necessary to commit MSCs to the osteoblastic lineage are RUNX2, also called core-binding factor subunit alpha-1, and osterix. Both these factors are essential for osteoblastogenesis and, in their absence, no osteoblasts can be formed. Other important factors are activating transcription factor 4 and bone morphogenic proteins (BMPs), which provide important signals that are essential for complete osteoblastogenic differentiation [57,58].

In culture, osteoblasts resemble the features of fibroblasts, except for the expression of RUNX2 and the bone-building protein osteocalcin (OCN). At the morphological level, osteoblasts differ from fibroblasts for the unique, osteoblast-specific feature represented by the production of a mineralized extracellular matrix [59]. In the bone microenvironment, as osteoblasts differentiate, they start to secrete the organic portion of the bone matrix (osteoid) and to produce hydroxyapatite crystals that are deposited into the organic matrix to form the bone’s mineralized structure. Ultimately, the osteoblast becomes enclosed in the mineralized matrix and differentiate into mature osteocyte, a star-shaped cell representing the most abundant cell type of mature bone [59]. This terminally differentiated bone cell is involved in many aspects of bone biology, from the regulation of bone metabolism through the expression of the negative regulator of bone mass sclerostin, to detection and transduction of mechanical load into biological responses, thus adapting bone shape to daily mechanical forces [60]. However, the exact role of osteocytes in bone homeostasis is not completely defined, as reviewed by Prideaux et al. [61].

The first report of a possible role for galectin-3 in osteoblast differentiation came from Aubin’s laboratory in 1995. Using immunohistochemical and molecular approaches, this research group demonstrated that osteoblasts and hypertrophic chondrocytes share the expression of galectin-3, in addition to other markers such as alkaline phosphatase (ALP), OCN, bone sialoprotein (BSP), osteopontin (OPN), and collagen type I. In addition, they observed that, in cultures of fetal rat calvaria, differentiation of osteoprogenitor cells into mature osteoblasts forming bone nodules is preceded by a phase of intense cell division, followed by loss of proliferative capacity and progressively increased expression of the osteoblast markers mentioned above, including galectin-3 [31].

Subsequently, the same group confirmed and extended the initial observations by investigating galectin-3 (both protein and mRNA) expression and regulation in several osteoblastic model systems, including an in vitro model of osteogenesis and bone tissue in vivo [41]. The most striking evidence in favor of a key role of galectin-3 in osteogenic differentiation was the observation that while galectin-3 mRNA levels increased during long-term culture in rat calvaria cells, the mRNA expression of the lectin fell down with time in culture of rat skin fibroblasts. Moreover, the amount of galectin-3 mRNA and protein increased with time concomitant with the increase of osteoblast differentiation markers and bone nodules formation. Finally, a role for galectin-3 in osteoblast differentiation was also supported by the observations that (a) galectin-3 expression in differentiating osteoblasts was modulated by two hormones known to affect osteogenesis, such as glucocorticoids and 1,25-dihydroxyvitamin D; and (b) mature osteoblasts and osteocytes of calvaria bone showed strong galectin-3 positivity, while faint staining was detected in immature osteogenic cells residing in periosteum [41]. Although these early studies clearly demonstrated that galectin-3 is expressed by osteoblast and that its levels increase during osteogenesis in parallel with the increased expression of established osteogenic markers, the mechanisms by which galectin-3 may regulate differentiation and activity of osteoblast or bone metabolism remains to be determined. Surprisingly, no further studies have been conducted to pursue this aim. The only additional information on the role of galectin-3 in osteoblast biology has come from a study investigating the molecular mechanisms through which the mechanical load is converted into molecular signals in bone. Briefly, galectin-3 expression was found to be up-regulated in the human osteoblastic HOBIT cell line exposed to extracellular nucleotides concomitant to RUNX2 DNA-binding activity, thus suggesting that ATP and/or UTP released by osteocytes in response to mechanical stimuli may account for the mechanosensory function of these cells [62]. These data also confirmed indirectly the finding by Stock et al. that galectin-3 is a RUNX2 target gene in bone [32].

Though these studies suggest that galectin-3 is critical for osteoblast differentiation and function, a recent report showed that exogenous recombinant galectin-3 inhibited terminal differentiation of a human pre-osteoblast cell line [63]. This finding may suggest different, or even opposite effects of galectin-3 on osteoblastogenesis, depending on its intracellular or extracellular localization, as already reported for other important regulatory functions in different cell types [20].

Regarding the possible involvement of galectin-3 in osteoblast and bone pathology, a couple of studies investigated the AGE-receptor role of this lectin in the deleterious effects exerted by AGEs on osteoblasts, as the possible molecular mechanism implicated in the pathogenesis of bone remodeling disorders associated with aging and diabetes. These in vitro studies confirmed that galectin-3, also known as AGE-receptor 3, and the best-characterized AGE receptor RAGE, are both expressed in osteoblastic cell lines, including the MC3T3E1 mouse calvaria-derived osteoblasts, and showed that AGEs are able to induce mRNA and protein levels of both AGE receptors [43,44]. In a previous study, the same investigators showed that exposure to AGEs elicits a biphasic response in osteoblasts, with an early increase in cellular proliferation and expression of differentiation markers, followed by induction of apoptosis and reduced differentiation in longer exposure to AGEs [45]. Interestingly, the effect of AGE exposure on galectin-3 and RAGE up-regulation was also time-dependent, with a prompt induction of galectin-3 expression and a delayed increase of RAGE levels, which coincided with the opposite biological effects exerted by AGEs in osteoblasts [44]. Therefore, galectin-3 could be involved in the early effects of AGEs on osteoblasts, i.e., proliferation and differentiation, while RAGE could be responsible for the long-term harmful effects of these by-products, such as radical oxygen species (ROS)-induced apoptosis, activation of the Extracellular Signal-regulated Kinase signal transduction pathway and inhibition of differentiation. These results are also consistent with the opposite effects exerted by galectin-3 and RAGE in AGE-mediated disease conditions, with the former playing a protective role through clearance of these toxic by-products via endocytosis, and the second implicated in tissue injury [20,64]. Altogether, these findings point to a possible role of the AGE/RAGE axis in the disorders of bone remodeling associated with aging and diabetes, with galectin-3 as a putative protecting factor through scavenging of AGEs and promotion of osteoblast differentiation and function.

### 3.2. Galectin-3 in Osteogenic Differentiation Capacity of Mesenchymal Stem Cells

MSCs are important for tissue and organ regeneration, including the bone. Consistently, it has been reported that MSC commitment towards the osteogenic phenotype decreases with age, an observation that might partly explain the impaired osteoblastogenesis observed in age-associated osteoporosis [65,66]. However, though the number of mature osteoblasts is progressively decreasing with advancing age, it was reported that the number of MSCs in mature bone remains constant throughout life [67]. This suggests that the age-dependent gradual reduction of ostoblastogenesis might be the consequence of a loss of function of MSCs or, alternatively, a perturbation of the environmental signals involved in the osteogenic commitment of MSCs [46], such as growth hormone [68] and estrogens [69].

Interestingly, it has been recently demonstrated that exposure to the systemic environment of young, but not elderly individuals favors MSC functionality and bone repair through modulation of β-catenin [70]. Therefore, attention has recently focused on the search for secreted circulating factors favoring stem and progenitor cell function. In this endeavor, special consideration has been paid to extracellular vesicles (EVs), which are small vesicles carrying proteins, mRNAs, and other molecules through the circulation [71]. EVs are released by most of the body’s cells [72] and their cargo is delivered to specific recipient cells [73]. Recently, Weilner et al. demonstrated that EVs isolated from young donors were more effective in inducing osteoblastogenesis in vitro compared to vesicles derived from elderly individuals. Interestingly, this osteoblastogenic donor age-dependent effect mediated by EVs was directly associated with galectin-3 content, which was higher in the EVs from young individuals [46]. These data indicated that galectin-3 not only plays an important role in the late stage of osteoblast differentiation and maturation, as previously shown [32,41], but also enhances the osteogenic differentiation capacity of MSCs favoring β-catenin activity. Mechanistically, galectin-3’s serine-96 phosphorylation site was found to compete for the glycogen synthase kinase-3β phosphorylation site on β-catenin, which represents a crucial step in the degradation of this transcriptional coactivator [46]. Finally, Weilner et al. also showed that even a moderate increase of galectin-3 expression induced ALP and RUNX2 expression in MSCs, thus suggesting that galectin-3 is not only a target gene, but it is also a positive regulator of the master osteogenic regulator RUNX2 [46].

In conclusion, these data confirm and expand previous reports on the importance of galectin-3 in osteoblast differentiation and propose an additional role for this lectin in bone remodeling, demonstrating that MSCs osteogenic differentiation is dependent on galectin-3 levels in circulating EVs. Therefore, it is tempting to speculate that the age-dependent reduction of galectin-3 EVs content might contribute to the reduction of bone formation, development of osteoporosis, and increased facture risk in the elderly [46].

### 3.3. Galectin-3 in Osteoclast Biology

Osteoclasts are large multinucleated cells responsible for bone resorption by degrading mineralized matrix. This cell type is critical for normal bone remodeling and is responsible for bone loss in pathologic conditions by increasing resorptive activity. Osteoclasts are terminally differentiated cells from immature hematopoietic monocyte/macrophage progenitors, a process regulated by multiple factors, including receptor activator of NF-κB ligand (RANKL), its decoy receptor osteoprotogerin (OPG), and macrophage colony-stimulating factor released by osteoblasts [74]. Once recruited to the bone, osteoclast precursors crawl to the resorption site, fuse with other precursors, and attach to the bone surface. Attachment to the bone matrix is a critical event in osteoclast activation, leading to osteoclast actin cytoskeletal reorganization and formation of sealing zones, which are composed of specialized integrin-mediated adhesive structures called podosomes [75]. It is well-known that protein-bound carbohydrates on the outer cell surface contribute to specific cell-cell and cell-matrix recognition [47]. Therefore, receptors for carbohydrates on osteoclasts and their precursors likely have a pivotal role in mediating cell-matrix interactions and, possibly, in regulating other related activities, such as migration, differentiation, and bone resorption. Since galectin-3 has been recognized as an abundant laminin-binding protein of macrophages [76], which share with osteoclasts a common lineage, its possible function in osteoclast activity has attracted some attention.

Galectin-3 expression by osteoclasts was originally described by Niida et al. in 1994. They reported that mouse tartrate-resistant acid phosphatase (TRAP)-positive mononuclear precursors (pre-osteoclasts) and mature osteoclasts are positive for galectin-3, but negative for the specific murine macrophage marker F4/80. In more detail, galectin-3 was detected in the cytoplasm and nucleus of pre-osteoclasts, as well as on their plasma membrane, suggesting a potential role of this lectin in cell-matrix adhesion during differentiation [48]. These initial findings were confirmed by Colnot et al. who reported that mature resorbing cells present an immunostaining for galectin-3 in their cytoplasm, including the multinucleated osteoclasts actively involved in resorbing the calcified cartilage core in the metaphysis and those present in the trabecular bone of neonatal mice [1]. They also noted positive staining for galectin-3 in mononuclear cells inside the bone marrow cavity [1]. Therefore, from these initial reports, it appeared that galectin-3 is expressed by bone osteoclasts and by their monocyte progenitors. Since galectin-3 is a well-known macrophage marker and is essential for phagocytosis [77], this lectin can be considered as a marker of the entire monocyte-macrophage-osteoclast cell lineage and it can be assumed that it is critical for some important osteoclast function. A few years later, Gorski et al. found two new alternatively spliced sequences of galectin-3 expressed by chicken osteoclasts [47]. Interestingly, galectin-3 expression was found to rise dramatically in bone marrow cells after 5 days of in vitro culture in an osteoclast differentiation media, suggesting a role for this lectin in the acquisition of the main phenotypic characteristics of osteoclasts, such as displaying TRAP activity, multinuclearity, and owning the capacity to resorb bone [47].

The role of galectin-3 in regulating osteoclast differentiation and activity was also investigated by Ortega et al. These Authors found that, besides playing a role in hypertrophic chondrocyte differentiation, galectin-3 also regulates osteoclast recruitment to the primary ossification center during endochondral bone formation, as well as differentiation of mononuclear osteoclast progenitors and osteoclasts survival at the chondro-osseous junction [40]. Consistent with a key role for endogenous galectin-3 in the biology of cells of the osteoclast lineage, Ortega et al. reported that mice null for MMP-9, the prominent MMP for cleavage of the N-terminal domain of galectin-3, displayed an increased number of galectin-3 positive cells, in parallel with an increase of TRAP immunostaining at the front of ossification. They also confirmed these data in an embryonic metatarsal culture system, observing that an excess of exogenous recombinant galectin-3, besides from increasing the number of TRAP-positive cells at the front of ossification, also interfered with recruitment of osteoclast precursors. Altogether, these findings suggest that aberrant persistence of higher levels of extracellular galectin-3, both exogenous or as a consequence of reduced cleavage by MMP-9, may induce abnormal osteoclast survival and, eventually, excessive bone remodeling [40].

### 3.4. Galectin-3 in Bone Remodeling

Bone remodeling is a lifelong process by which bone is renewed to maintain bone mass. Bone remodeling involves finely orchestrated cellular and molecular events and is commonly viewed as a two-step process carried on by bone cells: it involves old bone resorption by osteoclasts followed by new bone formation by osteoblasts [78]. These two arms of bone remodeling must occur in a balanced and coordinated manner in order to maintain bone mass and shape largely unchanged throughout adult life. Therefore, both increased bone resorption by osteoclasts or reduced bone formation by osteoblasts can lead to bone loss [79,80]. Indeed, bone metabolism is more complex than a two-step process, and there is still a lot to be learned about cellular and molecular mechanisms involved in remodeling. Particularly, unbalance between bone resorption and bone formation leading to reduced bone mass might be the result of multifaceted events affecting simultaneously osteoblasts and osteoclasts. Unfortunately, our knowledge of the molecular regulators that modulate differentiation and activity of osteoclasts and osteoblasts is still insufficient [81], thus hampering the identification of new therapeutic strategies to reduce the health burden and costs related to osteoporotic fractures, which are expected to increase in the future [82].

Although galectin-3 has been found to play a critical role in the differentiation and/or function of chondrocytes, osteoblasts osteocytes and osteoclasts [1,31,32,36,37,38,39,40,41,42,44,45,47,48,62,63], and even in favoring MSC osteogenic differentiation [46], so far, only one study has addressed the role of this lectin in bone homeostasis [49]. Simon et al. demonstrated that *Lgals3*^−/−^ mice exhibited altered bone homeostasis, as attested by histomorphometric analysis and micro-computer tomography measurements revealing a decreased trabecular bone volume in 12-week-old male *Lgals3*^−/−^ mice compared to wild-type littermates. Surprisingly, however, the role of galectin-3 in osteoblast differentiation was not investigated in this study, nor it was evaluated the effect of galectin-3 deficiency on the ability of osteoblast to form calcified nodules in vitro and new bone (bone formation rate) in vivo. However, at this age, the number of osteoblasts in bones of *Lgals3*^−/−^ mice was not different compared with wild-type mice [49]. At variance, bones of *Lgals3*^−/−^ mice exhibited an increased number of osteoclasts in the absence of changed RANKL/OPG ratio, a finding suggesting a direct inhibitory effect of galectin-3 on osteoclastogenesis. Consistent with this hypothesis, but at variance with previous studies indicating a positive effect of galectin-3 on osteoclast differentiation and maturation [40,47,48], bone marrow cells from *Lgals3*^−/−^ mice displayed an increased osteoclastogenic ability in ex vivo differentiation assays combined with a higher resorption activity. From a mechanistic perspective, molecular analysis revealed higher mRNA levels of the tumor necrosis factor receptor associated factor 6, a critical factor in RANKL signaling and terminal differentiation of osteoclast progenitors. Also in contrast to previous studies [40,47,48], exogenous galectin-3 added to wild-type murine or human osteoclasts was able to inhibit osteoclast differentiation. Moreover, co-culture assays of wild-type osteoclasts with galectin-3 deficient osteoblasts favored osteoclast maturation (size) and number [49]. Based on these findings, Simon et al. concluded that galectin-3 may be part of a molecular mechanisms through which osteoblasts control osteoclastogenesis at sites of mineralization. However, further research is needed to characterize the bone phenotype of *Lgals3*^−/−^ mice during growth and aging, to clarify the role of endogenous galectin-3 in osteoclast differentiation and function and, importantly, to elucidate the role of this lectin in regulating osteoblast activity in vivo. Regarding this last topic, previous reports described irregular structure of the extracellular matrix in bones from *Lgals3*^−/−^ mice, which displayed loss and reduced length of collagen fibers compared with wild-type animals [42]. Interestingly, these structural abnormalities of the bone matrix may account for the empirical observation of bone femur fragility of these mice [83]. Altogether, these findings suggest a critical role for galectin-3 also in the molecular architecture of the mineralized matrix, in maintaining bone quality, and in the optimization of bone strength. However, these assumptions were not confirmed by Simon et al. who reported unchanged mechanical stiffness of femurs in *Lgals3*^−/−^ mice compared with wild-type mice, as assessed by four-point bending test [49].

## 4. Galectin-3 in Inflammatory Bone and Joint Disorders

Arthritis is an inflammatory disorder of the joints. The two most common form of arthritis are rheumatoid arthritis (RA) and osteoarthritis (OA). They share common symptoms, such as pain, swelling, and stiffness of the joints, leading to disability. However, these two inflammatory joint disorders differ in etiological factors and pathogenesis [84]. In fact, while RA is an autoimmune disease characterized by autoantibody production, no specific causes have been identified for OA, which is generally considered a degenerative disease, being the result of “wear and tear” of the joints. Moreover, though chronic inflammation is a feature of both joint diseases, synovitis is a primary event in RA [85], whereas inflammation of the synovial membrane is a late event in OA, secondary to cartilage destruction and erosion [86]. In the clinical setting, synovial levels of galectin-3 are elevated in both RA and OA [87,88,89], though to a greater extent in the former [87,88]. However, it is unclear how galectin-3 is involved in the pathogenesis and progression of RA and OA, also because of the contradictory results obtained so far from diverse studies on different experimental models of these disorders. For the purpose of the review, we will only discuss in detail the most important experimental studies on the role of galectin-3 in OA and RA and the reader is referred to a recent review on the subject [84].

The role of galectin-3 in articular tissues and OA has been recently investigated using human OA chondrocytes and mice with OA induced by injection of mono-iodoacetate (MIA). Besides confirming the critical role of galectin-3 in chondrocyte survival [1,32,36,37,40,42], this study demonstrated that intracellular galectin-3 preserves cartilage structures, as evidenced by the finding of an accelerated age-dependent cartilage erosion in *Lgals3*^−/−^ mice. In fact, cartilage score of 4-month-old *Lgals3*^−/−^ mice was the same as in coeval wild-type mice injected with MIA, which also displayed cartilage lesions similar to those found in *Lgals3*^−/−^ mice. Moreover, the decrease of subchondral bone surface induced by MIA in wild-type mice was further accentuated in *Lgals3*^−/−^ mice, suggesting that galectin-3 may be essential for remodeling of the subchondral bone. Thus, this study showed a protective role of intracellular galectin-3 in OA and suggested that it is essential for cartilage homeostasis during ageing as its absence induced OA-like lesions. In addition, lack of galectin-3 during the OA process accelerated tissue injury in the joint [90].

In RA, the chronic inflammation of the synovial lining is accompanied by severe destruction of articular cartilage and bone erosion [85], which is mainly mediated by osteoclasts [50,91]. The prominent role of osteoclasts was also demonstrated using mice deficient in RANKL, an essential factor for osteoclast differentiation, showing that they were protected from articular bone loss in a serum transfer model of arthritis [92]. However, to make things more complicated, osteoblast function and new bone formation may also be negatively affected at sites of inflammation and erosion, thereby contributing to the net loss of bone [93]. Li et al. investigated the involvement of galectin-3 in bone destruction around the ankle joints in rats with adjuvant-induced arthritis (AA), which is considered an experimental model of human RA [94]. In this study, galectin-3 was shown to be abundantly expressed by macrophages and granulocytes accumulated in the areas of severe bone destruction in rats with AA, but only weakly by osteoclasts. This observation indicated an association between galectin-3 expression and incidence of arthritis. Moreover, galectin-3 injection in the affected joints was able to suppress bone destruction and reduce osteoclast recruitment, suggesting a protective role of the lectin. In vitro experiments confirmed the in vivo findings, demonstrating that exogenous recombinant galectin-3 was able to inhibit both formation of osteoclasts and dentine resorption by mature osteoclasts. Therefore, this study demonstrated a protective role of galectin-3 in AA-induced bone destruction and suggested that the protective effect of galectin-3 may be mediated by its negative regulation of osteoclastogenesis [94]. However, this conclusion is in contrast with previous data suggesting a critical role for this lectin in osteoclast differentiation and function [40,47]. Moreover, from these data, it cannot be ruled-out a protective role mediated by the anti-inflammatory, pro-resolution effect of galectin-3 [20,27,28,29,30,95]. Likewise, it cannot be excluded the possibility that the pro-osteogenic properties of galectin-3 may have contributed to protection from net bone loss. It must also be noticed that a recent work has put into question the results obtained by Li et al., proving that *Lgals3*^−/−^ mice are protected from joint injury associated with AA [96].

To our knowledge, the only study on the role of galectin-3 in a specific, non-tumoral, bone pathology was performed by Wehrhan et al. who investigated the molecular mechanisms involved in the pathogenesis of bisphosphonate-associated osteonecrosis of the jaw (BRONJ). BRONJ is an oral disorder characterized by an impairment in hard- and soft tissue repair. It is characterized by bisphosphonate-related impairment of oral tissues remodeling, involving bone cells, fibroblasts, and keratinocytes [97]. In addition, Reid et al. reported that BRONJ was associated to increased osteogenic differentiation of mucoperiosteal progenitors, anergy of the affected tissues, and increased galectin-3 expression in the periosteum and fibrous tissue stroma cells [97], thereby differing from osteoradionecrosis-related mucoperiosteal tissue, which was characterized by local inflammation and significantly less galectin-3 staining [98]. On the basis of the evidences indicating that galectin-3 is involved in the differentiation of osteoblasts and chondroblasts [1,31,32,36,37,40,41,42,44,45] and mediates inhibition of intraoral inflammation [97,99], the Authors concluded that the higher levels of galectin-3 might be involved in both the increased osteogenic differentiation of the mucoperiosteal precursors and the inflammatory anergy observed in BRONJ-affected soft tissues.

## 5. Galectin-3 in Vascular Osteogenesis

A large body of evidence indicates that vascular calcification is an unfavorable event in the natural history of the atherosclerotic disease. Consistently, coronary calcium score independently predicts cardiovascular morbidity and mortality, thus directly linking the amount of calcium in the vessel wall with unfavorable outcomes [100,101]. However, recent findings suggest that vascular calcification may either promote plaque progression and vulnerability or favor plaque stability, depending on the elemental composition [102] and pattern [33] of calcium deposition within the vessel wall. For instance, intravascular ultrasound studies indicate that spotty, granular and less dense calcification (commonly referred to as “microcalcification”) within a fibro-atheromatous plaque is the main pattern of calcium deposition in patients with acute myocardial infarction, whereas extensive dense calcification (commonly referred to as “macrocalcification”) prevails in subjects with stable angina pectoris [103,104,105]. However, despite the increasing body of evidence supporting the dual role of calcification in plaque stability and its clinical implications, little is known about the biological mechanisms that favor one type of vascular calcification over the other.

Though previously considered as a passive degenerative phenomenon [106], vascular calcification is now proven to be an active, tightly regulated process, similar to bone mineralization, activating an osteogenic gene regulatory program and involving various bone-related proteins. Many mechanisms have been proposed to explain the phenomenon of ectopic calcification within the vessel wall, including the osteogenic transdifferentiation of vascular smooth muscle cells (VSMCs) [107]. These specialized contracting cells are an important structural component of the artery wall, playing a key role in maintaining vascular tone and participating to various important functions, such as the regulation of blood pressure. VSMCs also play a key role in different pathological processes affecting the vasculature, including the pathobiology of vascular calcification. The process of VSMCs transdifferentiation towards osteochondrogenic precursors is a process characterized by progressive expression of the osteochondrogenic markers OCN, ALP, and OPN, associated with the expression of the osteochondrogenic transcription factor RUNX2. This process is also accompanied by phenotypic transition of VSMCs, attested by progressive reduction of expression of α smooth muscle actin, the main smooth muscle lineage marker, and loss of the contractile phenotype [108,109]. Several pro-inflammatory and pro-osteogenic stimuli have been shown to promote vascular calcification in the setting of atherosclerosis, including ROS [110], AGEs [111], advanced lipoxidation end-products [112], calgranulins [113], BMPs [114], and OPG [115]. However, most of the in vitro and in vivo studies performed to investigate the role of these stimuli in atherosclerotic vascular calcification have only assessed the quantitative aspect of the phenomenon, irrespective of the type of calcification process in which they are involved.

Some insight into the mechanism regulating the pattern of calcification in the process of atherogenesis has been recently provided [33]. Interestingly, this mechanism involves galectin-3 and RAGE, which were previously shown to modulate in opposite ways osteoblast differentiation and function in the presence of AGEs. In fact, while galectin-3 was shown to favor osteoblast differentiation, RAGE was found to be responsible for the detrimental effects of AGEs on osteoblasts, such as apoptosis and reduced differentiation [44]. Consistent with a pro-osteogenic effect of galectin-3 also in VSMC transdifferentiation, the increased expression of osteogenic markers induced by incubation in osteogenic medium was accompanied by a parallel increase in galectin-3 expression, in accordance with what was observed in differentiating osteoblasts [31]. In addition, galectin-3 deficiency was associated with defective osteogenic transdifferentiation of VSMCs, as attested by reduced expression of the osteogenic transcription factor RUNX2 and the bone cell marker ALP, enhanced apoptosis, reduced proliferation, and disorganized mineralization [33]. *Lgals3*^−/−^ VSMCs cells also showed decreased activation of Wnt/β-catenin signaling, which is in agreement with galectin-3’s ability to stabilize and increase β-catenin levels in MSCs differentiating towards the osteogenic lineage [46]. Noteworthy, the reduced ability of *Lgals3*^−/−^ VSMCs to acquire an osteogenic phenotype was also associated with RAGE up-regulation, and was exacerbated by treatment with AGEs, once again in keeping with previous data on osteoblast differentiation [44]. In vivo analysis of carotid plaques from patients undergoing carotid endarterectomy supported the hypothesis that galectin-3 and RAGE regulate in opposite ways the pattern of calcification, with RAGE expression observed in plaque regions with diffuse microcalcification and galectin-3 being mainly expressed by VSMCs in regions adjacent to extended and dense macrocalcification, where it colocalized with the osteogenic marker ALP [33].

Altogether, these data demonstrated that galectin-3 is essential for full transdifferentiation of VSMCs into an osteoblast-like phenotype via modulation of the Wnt/β-catenin pathway. They also provided evidence for a role of galectin-3 and RAGE in determining the pattern of calcification in atherosclerotic lesions. Noteworthy, the roles played by galectin-3 and RAGE in regulating VSMC transdifferentiation and vascular calcification recapitulate the effects they have at the skeletal level. In fact, galectin-3 was shown to regulate chondroblast/osteoblast differentiation/function and bone development [1,31,41]. Conversely, RAGE was shown to be essential for osteoclast maturation and function in skeletal tissue both in vivo and in vitro [116].

Finally, an ancillary observation is that a large body of literature indicates a role of galectin-3 as a prognostic marker in cardiovascular disease. In particular, elevated levels of circulating galectin-3 have been found to be associated with higher risk of death in heart failure patients [117]. Moreover, some experimental evidence suggests an active role of this lectin in cardiac remodeling and atherosclerosis, mainly through its recognized fibrogenic properties [20,117]. However, given the important role played by vascular calcification in atherosclerosis and cardiovascular disease, and that of galectin-3 in VSMCs osteogenic transdifferentiation, it cannot be rule out the possibility that circulating galectin-3 might affect cardiovascular disease also through its osteogenic properties.

## 6. Conclusions

Most of the study conducted to investigate the role of galectin-3 in the biology of bone and its cells date back to the late 90s of last century and the beginning of 2000s. Although they produced convincing evidence about the involvement of this lectin in multiple biologic processes of the bone, such as differentiation and activity of all major bone cells, they were not followed by an in-depth analysis of the role of galectin-3 in the bone physiology and pathology. Only recently, the skeleton of *Lgals3*^−/−^ mice has been analyzed and results demonstrated that, in fact, adult (3-month-old) mice have a bone phenotype, characterized by reduced trabecular bone compared with wild-type mice [49]. However, the analysis of the mechanisms underlying the structural abnormalities only investigated the resorption arm of the bone remodeling process, leaving completely unexplored the bone deposition arm and the cells responsible for bone formation. Since, galectin-3 has been shown to have a critical role for chondroblast and osteoblast differentiation and activity [1,31,32,36,37,40,41,42,44,45], an in-depth analysis of these cells and their function in *Lgals3*^−/−^ mice is necessary to understand the net effect of this lectin on bone homeostasis. It would also be desirable to extend the analysis to the skeleton of younger and older *Lgals3*^−/−^ to get information on the role of this lectin in growth and age-dependent changes of the bony structure. These analyses could provide valuable information possibly helping in identifying galectin-3 as potential therapeutic target in some bone pathologies, primarily senile osteoporosis. This concept is strengthened by the evidence that, in addition to its role in osteogenesis, (a) galectin-3 is able to stimulate osteogenic differentiation of MSCs [46]; (b) in osteoblast lineage cells [43,44,45], it acts as a receptor capable of degrading AGEs, which have been implicated in age-dependent [118] and diabetes-associated [119] bone fragility; and (c) it may favor resolution of chronic inflammation [20], also in the bone tissue [20,97,98,99].

Finally, the osteogenic properties of galectin-3 deserve to also be investigated in the process of ectopic calcification, primarily vascular osteogenesis. The possibility of modifying the amount and especially the type of calcification in the atherosclerotic plaque by modulating the expression of galectin-3 could be very useful in stabilizing the atherosclerotic lesions, thus reducing the risk of fatal events.

## Figures and Tables

**Table 1:** Proven roles of galectin-3 in bone biology and supposed functions, to be investigated, in the pathophysiology of bone metabolism.

Cell	Proven	To Be Investigated
**Chondrocyte**	cell marker and pro-survival factor [1,36] regulator of cell-to-cell interaction with chondroclasts [37] target and regulator of MMPs activity [38,39,40]	mechanisms underlying the regulation of chondrocyte activity and survival
**Osteoblast**	RUNX2 target gene [32] differentiation marker [41] proper bone ECM structure by regulating collagen fibers synthesis [42] protection against AGE mediated toxic effects by its AGE-receptor scavenger activity [43,44,45]	mechanisms underlying the regulation of osteoblast differentiationprotection from age- and diabetes-related bone fragility
**Osteocyte**	cell marker [1,41]	mechanosensory function and promotion of bone modelling and remodeling
**MSC**	increased osteoblastogenic differentiation capacity [46] positive regulation of the master transcription factor RUNX2 [46] stabilization and increase of β-catenin levels [46]	promotion of bone repair and homeostasis through modulation of β-catenin
**Osteoclast**	differentiation marker [47] mediator of cell matrix adhesion [48] regulation of differentiation from progenitors and pro-survival factor [40] downstream regulator of MMP-9 activity [40] and ECM degradation [37] negative regulation of osteoclastogenesis [49,50]	opposite effects depending on intra- and extracellular localization

MMPs = matrix metalloproteinases; RUNX2 = Runt-related transcription factor 2; ECM = extracellular matrix; AGEs = advanced glycation endproducts.

## Abbreviations

AGEs	Advanced Glycation
RAGE	Receptor for AGEs
RUNX2	Runt-Related Transcription Factor 2
*Lgals3*^−/−^	Galectin-3 Null
MMPs	Metalloproteinases
MSC	Mesenchymal Stem Cell
BMPs	Bone Morphogenic Proteins
OCN	Osteocalcin
ALP	Alkaline Phosphatase
BSP	Bone Sialoprotein
OPN	Osteopontin
EVs	Extracellular Vesicles
RANKL	Receptor Activator of NF-κB Ligand
OPG	Osteoprotogerin
TRAP	Tartrate-Resistant Acid Phosphatase
RA	Rheumatoid Arthritis
OA	Osteoarthritis
MIA	Mono-Iodoacetate
AA	Adjuvant-Induced Arthritis
BRONJ	Bisphosphonate-Associated Osteonecrosis of the Jaw
